# Does Non-Immersive Virtual Reality Improve Attention Processes in Severe Traumatic Brain Injury? Encouraging Data from a Pilot Study

**DOI:** 10.3390/brainsci12091211

**Published:** 2022-09-08

**Authors:** Rosaria De Luca, Mirjam Bonanno, Carmela Rifici, Patrizia Pollicino, Angelo Caminiti, Giovanni Morone, Rocco Salvatore Calabrò

**Affiliations:** 1 Neurorehabilitation Unit, IRCCS Centro Neurolesi “Bonino Pulejo, 98124 Messina, Italy; 2 Department of Life, Health and Environmental Sciences, University of L’Aquila, 67100 L’Aquila, Italy; 3 Rehabilitation Centre, San Raffaele Institute of Sulmona, 67039 L’Aquila, Italy

**Keywords:** traumatic brain injury, attention process training, virtual-based cognitive rehabilitation

## Abstract

Traumatic brain injury (TBI) is a sudden injury that causes damage to the brain. Rehabilitation therapies include specific training, such as attention process training (APT) programs using either standard or innovative approaches. The aim of this study is to evaluate the effects of a non-immersive virtual reality-based attention training to stimulate attention processes and mood in TBI patients. Thirty subjects with TBI were enrolled at the Neurorehabilitation Unit of the IRCCS Neurolesi Center and divided into either the Conventional Attention Process Training Group (C_APT: *n* = 15) or the Virtual-Based Attention Processes Training Group (VB_APT: *n* = 15), treated with the Virtual Reality Rehabilitation System (VRRS-Evo). All of the patients were evaluated with a specific psychometric battery before (T0) and after the end (T1) of each program. We found statistically significant differences between the two groups, in particular concerning global cognitive status (*p* < 0.02), attention processes (*p* < 0.03), depression symptoms (*p* < 0.04) and visual attention (*p* < 0.01). Experimental intragroup analysis showed great statistical significances in all psychometric tests, i.e., the Montreal Cognitive Assessment (*p* < 0.0006), Attention Matrices (*p* < 0.0007), the Hamilton Rating Scale-Depression (*p* < 0.004), the Trail Making Test-A (*p* < 0.0007), the Trail Making Test-B (*p* < 0.0007), and the Trail Making test-BA (*p* < 0.007). Our results suggest that non-immersive virtual reality may be a useful and effective approach for the attention processes recovery and mood of TBI patients, leading to better cognitive and behavioral outcomes.

## 1. Introduction

Traumatic brain injury (TBI) is one of the leading causes of morbidity, disability, and mortality, especially at young-adult age [1]. It is a damage to the brain caused by an external mechanical force, which determines permanent or temporary cognitive impairments in association with physical, psychosocial dysfunctions, and a diminished or altered state of consciousness [2]. It is estimated that approximately 5.48 million people suffer from severe TBI each year (73 cases per 100,000 people) [3,4]. Among the survivors of moderate to severe head injury, 31.8% of patients die or need hospitalization in a specialized health center, 44% are unable to return to work, and 88% of the patients with mild TBI have white matter damage, with negative repercussions on functional outcomes [5]. Indeed, TBI is usually identified as mild, moderate, or severe through diagnostic and prognostic clinical scales [6], such as the Glasgow Coma Scale, which measures the level of injury in relation to the loss of consciousness, memory loss, or type of responses to different levels of verbal or not stimuli [7]. The treatments for TBI depend on many factors, including the size, severity, and location of the brain injury [8,9].

TBI people can be characterized by motor dysfunctions and cognitive and behavioral alterations with important consequences on their quality of life. Cognitive deficits are among the concerns that negatively affect the quality of life of these individuals [10,11]. In particular, impairments of attention are very common and are expected to occur in approximately 80% of all TBI patients [12]. Moreover, it is increasingly recognized that these patients have co-occurring psychological and physical conditions that may impact cognition, especially sustained attention and executive functioning [13]. Growing evidence demonstrates that cognitive rehabilitation (CR) is effective in patients with TBI as it enhances cognitive and psychosocial interaction [14,15]. In their review, Michel and Mateer discussed various approaches to attention rehabilitation in individuals following stroke and TBI, demonstrating that Attention Process Training (APT) may be considered a standard practice in the post-acute phase, because it seems that attention recovery could be a critical modulator of neuroplasticity [16]. However, its ability to generalize to untrained abilities or functional capacity has yet to be conclusively demonstrated. The rehabilitation of attention deficits following the acquired brain injury is essential for a good reintegration in daily living and social and working life [17]. According to Bartfai et al., knowledge of prognostic factors, including the severity of attention deficit to maximize the efficiency of resource allocation and the effectiveness of rehabilitative interventions, is fundamental to enhance outcomes following TBI [18].

More in detail, cognition is not a unitary concept: it incorporates multiple domains, including attention (focused, shifted, divided, or sustained attention on a particular stimulus or task), and other related cognitive functions, and this is essential to stimulate a dynamic cognitive remediation for TBI patients [19,20]. Attention deficits may be improved using systematic and repetitive cognitive training. For these reasons, APT is considered a standard method in clinical practice, recommended with a high priority level [21,22], i.e., it should be regularly used to ameliorate attention deficits after brain injury [23,24,25]. Historically, the recovery of attention deficits in TBI has been carried out utilizing a restorative drill and practice approaches with visual or auditory stimulus–response paradigms, using a face-to-face setting with a paper and pencil approach [26]. Recently, various researches have shown that new neuropsychological treatment, focusing on virtual reality (VR) training, can be useful in TBI patients. In fact, VR has been used successfully to stimulate attention processes following TBI [27,28,29,30]. Besides, the current literature reported that information and communication technologies and PC-based solutions, such as serious games, software-dedicated CR, VR simulations, or other computer-mediated approaches, have an enormous impact to promote the intensity and personalization of APT, using ecological and motivational tasks [31,32]. Among rehabilitative virtual systems, the VRRS-EVO (Khymeia, Padua, Italy) has an intuitive and simplified interface thanks to “remote touch”, which allows the immediate management of all its functions, offering an augmented feedback to the patients. In fact, the VR offers the possibility to simulate daily activity in a non immersive virtual environment, adapting the task parameters according to the patient’s performance, which increases training specificity and patient’s motivation by avoiding boredom and frustration in a more sophisticated and ecologically valid approach [33].

The aim of this study is to evaluate the effects of a systematic non-immersive virtual-based attention training to stimulate attention processes (selective, alternating, sustained, and split) and mood in TBI patients.

## 2. Materials and Methods

Thirty patients affected by TBI in the post-acute/chronic phase (at least 3 months after the event), having attended from April 2021 to September 2021 the Outpatient clinic of the Neurorehabilitation Unit of the IRCCS Neurolesi “Bonino Pulejo” of Messina, were enrolled in this study. A more detailed description of the 2 groups is listed in Table 1.

Continuous variables are expressed as mean ± standard deviation, whereas categorical variables as frequencies and percentages. *p*-values referring to score tests are presented at the onset of the study (T0), confirming that groups were not statistically different at the beginning except for the MoCA’s abstraction subtest.

Either TBI patients and/or the relatives were adequately informed about the study and offered their collaboration and the written consent. The study was performed following the Helsinki declaration of human rights, and the local Ethics Committee approved the study (IRCCS-ME-CE 08/21).

The patients were randomly assigned to one of 2 groups using a web-based application for block randomization (www.randomization.com, accessed on 21 March 2022). In particular, we used the block randomization method (block size = 4) in order to ensure balance in the sample size across groups over time. The experimental group received the innovative attention training (VB_APT; 7 male patients and 8 female patients with a mean age of 44.6 ± 14.4 years), and the control group (C_APT; 7 male patients and 8 female patients, mean age 50.5 ± 17.9 years) was submitted to standard CR.

Inclusion criteria were: (i) diagnosis of first ever severe TBI in the post-acute/chronic phase, i.e., ≥3 months from the traumatic event; (ii) presence of moderate cognitive alterations following TBI (i.e.,) MoCA ≥ 16; and (iii) absence of disabling sensory alterations (i.e., hearing and visual deficit), severe psychiatric, and medical illness.

Patients were excluded if they presented severe cognitive and behavioral deficits potentially interfering with the training.

Each participant was evaluated by a blinded to treatment neuropsychologist, through the administration of specific neuropsychological tests. The evaluation was administered before (T0) and after (T1) both attention processes training.

The cognitive psychometric screening included the assessment of global cognitive function as well as specific neuropsychological tests to evaluate the attention processes abilities in different components (Table 2). Outcome measure were: (1) Montreal Cognitive Assessment (MoCA) [34], a rapid screening instrument to evaluate various neuropsychological sub items: attention processes, executive functioning, memory functions, language, visuo-constructional abilities, thinking, calculations, and orientation; (2) Attentive Matrices (AM) to evaluate the visual selective attention [35]; and (3) Trail Making Test (TMT) that measures attention process, visual search and scanning, sequencing and shifting, psychomotor speed, abstraction, and flexibility and executive functions [36]. The Hamilton Rating Scale for Depression (HRS-D) was administered to investigate the presence and level of depression symptoms [37], to avoid confounding factors in cognitive training and recovery.

All study participants underwent the same standard cognitive rehabilitation, 3 times a week for 8 weeks (i.e., 24 sessions of 45 min each). In addition, the experimental group was submitted to the VB_APT, using the VRRS system (24 sessions of 60 min each, 3 times a week for 8 weeks), while the controls performed the same amount of standard CR of the attention deficits (24 sessions, 3 times a week for 8 weeks) (see Table 3).

### 2.1. Conventional Attention Processes Training (C_APT)

According to Sohlberg and Mateer’s clinical model of attention [38], we have submitted the control group to a specific rehabilitative attention program consisting of a series of pencil and paper exercises using standard rehabilitative materials (imagines, colors, barrage task) and a to face-to-face approach with the cognitive therapist in a dedicate hospital room (a quiet environment, without disturbing noises or distractions).

The APT is a hierarchically-organized and individual-based attention-training program, which includes systematic tasks and is based on meta-cognitive strategy and psycho-education interventions [22]. Sohlberg and Mateer’s model was used as the framework for organizing the materials in the APT program. We have divided the exercises into five components: (1) sustained attention, (2) selective attention, (3) alternating attention, and (4) divided attention. The attention rehabilitative program was indeed organized for these specific domains, and included activities and task-oriented exercises, using visual cues, and work on auditory skills in three levels of difficulty.

### 2.2. Virtual Reality Based-Attention Processes Training (VB_APT)

**Figure 1:**
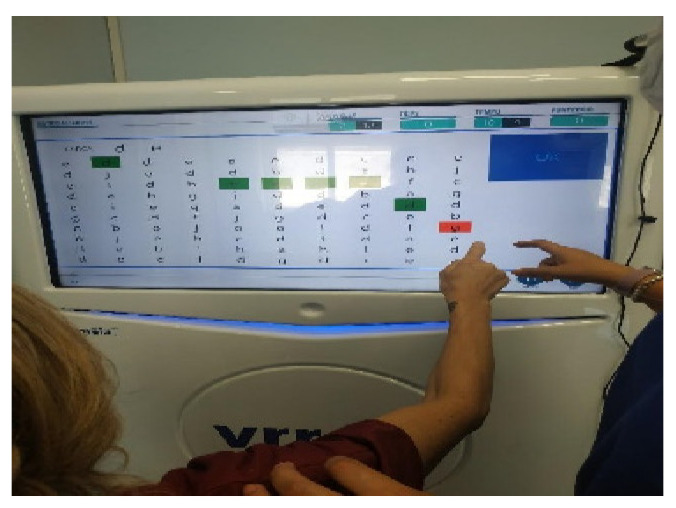
Shows the 2D VR training modality: Psychiatric therapist guided the patient in carrying out goal-oriented, manual-eye coordination exercises, while she is seated in a wheelchair in front of the VRRS station. In red, it is marked the wrong response.

**Figure 2:**
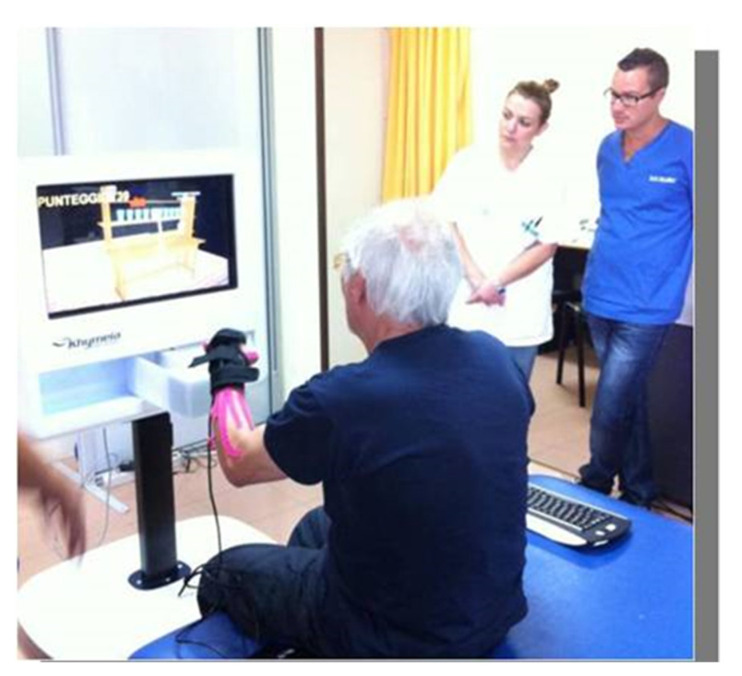
Shows the “3D” interaction modality (through the sensors located over the upper limb).

VRRS is one of the most advanced, comprehensive, and clinically tested virtual reality system for rehabilitation and tele-rehabilitation. VRRS, with the exclusive magnetic kinematic acquisition system, is used for the rehabilitation of a wide spectrum of neurological diseases via the numerous rehabilitative modules, including neurological, logopaedic, and cognitive ones. During the APT, the patient was sitting in front of the device, actively interacting with the device. In particular, the VRRS cognitive-motor integration module consists of a large set of interactive activities for attention rehabilitation, some specific oculo-motor coordination tasks, using virtual touch modality, with more than 50 exercises already available and many others under development to stimulate different attention’s processes, including specific attention sub-domains. Each type of virtual exercise provided through the VRRS can be organized in 2 main criteria, differing in the way of interaction with the virtual reality tool. The 1st category includes 2D exercises where the patient interacts with objects and scenarios through the touch screen or through a particular magnetic tracking sensor coupled with a squeezable object, thus emulating mouse-like interaction capabilities. The 2nd category consists of 3D exercises, where the patients interact with 3D on virtual scenarios and objects through magnetic wearable sensors generally placed over the hand (that permits a 3D position tracking of the end effector). This means that, although non-immersive VR is in both cases represented in the same flat screen, using such a “3D” modality, upper and lower limbs are able to move in the 3-dimensions of the space while interacting with the virtual environment (Figure 1 and Figure 2).

Furthermore, neuropsychological tasks consisted of pick and place activities, ordering activities, selection tasks, and sequential selection. The VRRS cognitive training was based on a game interaction, using augmented feedback with a repetitive stimulation of each attention sub item, such as selective attention, alternating, sustained, and split. Notably, the level of the exercise performed can be simple, moderate, and advanced in relation to satisfaction of specific criteria: execution’ time, number of repetition and errors in each set, and indicators of the accuracy task performed. In fact, the psychiatric therapist planned and organized all virtual exercises (after consultation with the neurologist), increasing the difficulty in relation to the time of execution and the category of activity.

### 2.3. Statistical Analysis

Statistical analyses were performed on R 4.1.3 (Vienna, Austria) [39] for Windows and interpreted at the two-tailed significance level of 0.05. The normality of outcome measures was assessed at each time point and for the two study groups separately with the Shapiro–Wilk test. Since our distribution was not normal, we used the Wilcoxon signed rank test for intra-group analysis to compare pre/post intervention scores for each group while the Mann–Whitney’s test was used for the analysis between the two groups (control and experimental) to compare them on their post-intervention scores, and also to verify there were not statistical differences between subjects at the onset of the study. In addition, linear weak correlation between HRS-D and AM was calculated with Spearman’s rank correlation coefficient. The distributions of the test statistics were transformed into effect sizes (ES), calculated with Cohen’s d test, to investigate whether achieved treatment effects have a sufficient clinical impact [40].

## 3. Results

All of the thirty TBI patients included in this study completed the training without side effects, including cybersickness. Comparing post-intervention scores, we found statistically significant differences in the results between the two groups, in particular in MoCA (*p* < 0.02), AM (*p* < 0.03), HRS-D (*p* < 0.04), and in TMT-A (*p* < 0.01).

Intragroup experimental analysis, comparing pre-(T0) and post-(T1) treatment, showed strong statistically significant differences in all psychometric tests: MoCA (*p* < 0.0006, ES = 1.46), AM (*p* < 0.0007, ES = 0.86), HRS-D (*p* < 0.004, ES = 0.34), TMT-A (*p* < 0.0007, ES = 0.28), TMT-B (*p* < 0.0007, ES = 0.56), and TMT-BA (*p* < 0.007, ES = 0.35) as reported in Table 4. In addition, our data showed a weak linear correlation between depressive symptoms (HRS-D) and attention processes (AM) rho = −0.39. In the control group, analyzing the test scores at the beginning (T0) and at the end (T1) of the conventional treatment, a significant improvement was found in MoCA (*p* < 0.02, ES = 0.43), HRS-D (*p* < 0.009, ES = 0.43), TMT-A (*p* < 0.01, ES = 0.25), and TMT-BA (*p* < 0.002, ES = 0.52), whereas AM (*p* = 0.47, ES = 0.07) and TMT-B (*p* = 0.40, ES = 0.06) did not reach statistical significance.

Notably, analyzing the specific MoCA’s subtests, we found a statistical significance only in the experimental group concerning executive visuo-spatial and attention, at intra-group analysis comparing pre- and post-treatment. On the other hand, the between- groups showed a statistically significant difference in the attention’s item, after the treatment.

Finally, the minimal clinical important difference (MCID) of three points was used for the MoCA [40]: a total of 13 subjects of 15 achieved the MCID in the treatment group, whereas in the control group a total of 3 subjects of 15 achieved this threshold.

## 4. Discussion

To the best of our knowledge, this is one of the few studies aimed at evaluating the effects of a non-immersive virtual reality-based attention training to stimulate cognitive functioning in TBI patients. In line with current literature, our data suggest that both C_APT and VB_APT interventions may increase attention abilities and mood in such patients. However, only in the EG, which received APT using the VRRS, we found a more significant improvement in specific attention subdomains, including visual attention, task switching, visual search speed, visuo-spatial scanning, speed of processing, mental flexibility, and rote memory, with a large ES. In the CG, we instead found an improvement in TMT-A and TMT-BA, and the ES was quite near to medium only in TMT-BA. This demonstrates that both conventional and innovative CR tools can be useful in improving cognitive function in patients with TBI, but such an improvement is potentially greater when VR is applied.

We have also found, in the experimental group, a negative correlation between the reduction of depression symptoms and an increase in attention selective processes (rho = −0.39), as confirmed by Himanen et al., which demonstrate that impaired sustained attention may be mostly related to depressive symptoms in patients with chronic TBI sequelae [42]. This result is important because depression and cognitive status (above all attention) are two fundamental determinants of the subjects’ participation to the proposed neurorehabilitation, as demonstrated in other neurological central conditions [43]. On the other hand, participation and depression are key factors for the efficacy of neurorehabilitation [44,45].

Furthermore, the role of the executive function and cognitive-motor recovery is more complex: the presence of intact executive function may favor a better recovery in the activities of daily living as well as a better social participation [46,47], whereas the recovery of executive functions is the result of a more effective rehabilitation. This latter aspect has been recently more and more emphasized, since neurorehabilitation is more based on functional task-oriented therapy (with or without modern technology and devices) [48], in which working memory, task switching, short-term memory for example, are key factors for re-learning mechanisms [49,50]. To this end, VR may potentiate relearning also acting on attentive processes, as shown in our study.

It is important to highlight that VR differs from non-VR (videogame-based) applications by many factors, such as the used device (i.e., head-mounted display or CAVE vs. a standard 2D monitor), and the technical qualities (i.e., the degree of immersivity and interaction). These aspects categorized two different rehabilitation approaches in terms of the level of ecological validity and in terms of the attention mechanisms involved, resulting from the sensory–motor interaction between the user and virtual environment [51]. In addition, regarding immersive and non-immersive VR, it should be noted that there is no better or more advanced approach than another, but there are probably different rehabilitation methods to be used depending on the motor and cognitive impairment of the patient.

In our study, the non-immersive VR approach using the VRRS has been developed to both increase motivation during the neurorehabilitation training thanks to its enjoyable and interactive modality and potentiate the level of attention as well as the global cognitive recovery and psychological well-being [52,53]. Thanks to the VRRS training, individuals with TBI people receive augmented feedback to the central nervous system through tasks performed in a virtual setting serving to develop the knowledge of the results of movements (knowledge of the results) and the knowledge of the quality of movements (knowledge of performance), leading to a training-specific motor learning/relearning [54,55]. This enhancement in neural plasticity with a subsequent potentiation in the clinical measures could explain why our TBI patients attained higher results after this advanced VB_APT than following the C_APT. Similar positive results on motor and cognitive function have been found in other neurological disorders using the VRRS in either “in-hospital” or “home modality” (i.e., telerehabilitation) [56,57,58,59]. This is one of the main reasons why we decided to apply the device also in TBI patients.

Our study has some limitations to acknowledge. The sample is relatively small to extend the results to the general TBI population; however, the study was conceived as a pilot study and the number of subjects enrolled are in line with the study design. Another limitation was the allocation procedures that were not concealed, this should increase the risk of bias. Finally, it should be interesting to evaluate the effect of the present attention training on motor outcomes.

More studies with larger samples, higher quality methodology, and long-term follow-up, are needed to confirm the therapeutic effect of VR on cognitive and behavior outcomes.

## 5. Conclusions

Cognitive rehabilitation using VR, such as the VRRS, could be useful in optimizing attention processes recovery and reducing depression symptoms in post-acute TBI patients. This could therefore be considered as a complementary treatment for cognitive deficits in these vulnerable individuals and its use, once confirmed by larger sample studies, should be implemented in clinical practice.

## Figures and Tables

**Table 1:** Demographic and clinical description of the sample at the beginning of the study.

	Experimental	Control	All	*p*-Value
Participants	15	15	30	
Age	44.6 (±14.44)	42.53 (±17.95)	43.56 (±16.04)	0.86
Gender				
Male	7 (46.6%)	7 (46.6%)	14 (46.66)	
Female	8 (53.33%)	8 (53.33%)	16 (53.33%)	
Education				0.41
Elementary school	2 (13.33%)	4 (26.6%)	6 (20%)	
Middle school	2 (13.33%)	4 (26.6%)	7 (23.33%)	
High school	9 (60%)	4 (26.6%)	13 (43.33%)	
University	2 (13.33%)	3 (20%)	5 (16.66%)	
MoCA	22 ± 2.90	23.26 ± 3.69	22.63 ± 3.32	0.17
Executive Visuo-Spatial	3.13 ± 0.83	3.6 ± 0.98	3.6 ± 0.92	0.25
Denomination	2.93 ± 0.25	2.73 ± 0.45	2.83 ± 0.37	0.36
Attention	1.33 ± 0.87	1.48 ± 0.81	1.41 ± 0.84	0.45
Language	1.03 ± 0.85	1.16 ± 0.74	1.1 ± 0.79	0.58
Abstraction	1 ± 0.37	0.53 ± 0.51	0.76 ± 0.50	0.04
Deferred recall	3.2 ± 1.01	3.8 ± 1.08	3.5 ± 1.07	0.14
Orientation	5.66 ± 0.48	5.8 ± 0.41	5.73 ± 0.44	0.54
AMT	36.06 ± 10.54	34.25 ± 13.65	35.14 ± 12.02	0.56
HRS-D	10.53 ± 6.35	11.8 ± 4.41	11.16 ± 5.41	0.90
TMT-A	76.46± 50.83	121.8 ± 97.24	99.13 ± 79.65	0.15
TMT-B	206.86 ± 86.41	221.13 ± 111.67	214 ± 98.38	0.95
TMT-BA	127 ± 74.05	153.6 ± 142.24	140.3 ± 112.24	0.88

Abbreviations: MoCA, Montreal Cognitive Assessment, AMT, Attentive Matrices test; HRS-D, Hamilton Rating Scale for Depression; TMT-A, Trail Making Test-A, Trail Making Test-B; Trail Making Test-B-A.

**Table 2:** Description of cognitive assessment tools used in traumatic brain injury patients.

Test/Scale	Domains	Description
Montreal Cognitive Assessment (MoCA)	Global Cognitive status	The MoCA evaluate several neuropsychological sub-items: (1) Memory abilities (2) Visuo-spatial Skills (3) Executive Functions (4) Attention processes/working memory capacity (5) Verbal Fluency and Communication Skills (6) Abstract reasoning (7) Orientation to time and place MoCA scores range between 0 and 30. A score of 26 or over is considered to be normal.
Attentive Matrices (AM)	Attention Processes	The Attentive Matrices is used to evaluate the selective visual attention. There are 3 matrices that are shown to the subject. Each of them consists of 13 lines of 10 numbers from 0 to 9 each, arranged in a random sequence. The subject must block all numbers equal to those printed at the top of the matrix. Matrices should be presented from the simplest to the most difficult. The number of correct answers is calculated (range 0–60 overall in the three matrices); the number of false alarms (range 0–270 overall in the three matrices); omissions (range 0–60). The cut-off is ≤30
Hamilton Rating Scale-Depression (HRS-D)	Mood	HRS-D is a clinical scale used to evaluate the presence or not of the depression symptoms. It is articulated in 21-items, including 4 items intended to subtype the depression, but which are sometimes, incorrectly, used to rate severity. HRS-D is characterized by a specific scoring, from a not depressed: 0–7 to a very severe depressive status (severe): >23.
Trail making Test (TMT-A; TMT-B; TMT-BA)	Attention and visuo-spatial function	TMT assesses spatial planning capability in a visuo-motor task. TMT is composed of two parts, A and B. The trail A evaluates the sustained attention, the trail B measures split and alternate attention, and the difference in time between the 2 tests (B–A) is a factor of cognitive flexibility and shifting ability

**Table 3:** TBI-Attention Processes training, including both conventional and innovative training.

**Cognitive Domain**		Interaction	*C_APT Human interface*	*VB_APT VRRS evo System*
		Modality	Paper and pencil task	Pc-based task
			Face to Face setting	Human–web interface
			Direct interaction	Virtual interaction by VRRS
			3 levels of complexity for execution’s time and the numbers of stimuli-target and distractores administered	3 levels of difficulty for execution’s time and the numbers of stimuli-target and distractores administered
		Sub-items	*CAPT-Task*	*VRRS-APT Task*
**Attention Processes**	Execution Time 15 min For each attention component	Selective	Pointing the stimulus target and ignoring the distractor symbols. Locating the target symbol and ignoring the distractions; to indicate and touch directly with his hand the selected/standard target-stimuli in relation to specific characteristics presented (color, image, animals, function…) neglecting the distracters, which consist in other picture, different for number and complexity of criteria. Cognitive therapist showed the verbal commands to the patient, which combined the different selective images. The patient touches the standard target stimuli presented in a specific time, according to the therapist’s verbal command.	Scanning the entire screen to locate all of the information. To administer scanning exercise, the user must locate the target symbols in a grid, and select the matching virtual symbols. To select and immediately recall feedback (audio and video) similar to various elements: colors, musical strings, geometric or not form, animals…) observed in the virtual environment. The patient touches the virtual target element in a specific time, this action causes a visual change with a specific audio feedback (positive reinforcement), using VVRS-interaction between the cognitive therapist and patient. Otherwise, the element disappears (negative reinforcement).
		Alternating	Switching between stimulus A and stimulus B. To increase the attention alternating processes, the cognitive therapist organized specific activities, involving the mental flexibility for moving between tasks with different cognitive requirements, which use pencil-and-paper tasks (such as to make simple sequences of animals, fruits, objects-colors-pictures).	To increase the attention to alternating processes, the cognitive therapist selected specific virtual activities, involving the mental flexibility for moving between tasks with different cognitive requirements, which use computer games/software dedicated (such as to make simple sequences of animals, fruit, objects-colors-pictures).
		Sustained	To stimulate sustained attention processes, the patient observed different stimuli for a variable and progressive time, with an attentional focus on traditional tasks.	To stimulate sustained attention processes, the patient observed from 3 to 5 targets-stimuli for a variable and progressive time (10–15 min), with an attentional focus on virtual tasks.
		Split	The therapist asks the TBI patient to perform a double task such as selecting/associating the color to the shape and at the same time eliminating the different standard stimuli.	The therapist asks the TBI patient to perform a double task such as selecting/associating the color to the shape and at the same time eliminating the different shapes/virtual stimuli.

Legend: C_APT, Conventional Attention Process Training, VB_APT, Virtual Based Attention Process Training, VRRS, Virtual Reality Rehabilitation System.

**Table 4:** Median and first–third quartile of the administered psychometric tests in TBI patients, between virtual-based approach with VVRS and the conventional one. Wilcoxon signed the rank test for the intra-group analysis and Mann–Whitney’s test of neuropsychological evaluation between two groups. Significant *p*-values are in bold.

Psychomethic Tests	Attention Training	*p*-Value (Intha-Group Analysis)	Range (First–Third Quartile)	Median	*p*-Value (Between-Group Analysis)	ES
MoCA	VB_APT	**0.0006**	24.5–28.5	27	**0.02**	0.46
MoCA	C_APT	**0.02**	23.5–27	25		
Executive Visuo-Spatial	VB_APT	**0.001**	1–5	5	0.33	0.43
Executive Visuo-Spatial	C_APT	0.12	3–3	2		
Attention	VB_APT	**0.004**	1–2	1	**0.04**	0.44
Attention	C_APT	0.24	1–2	1		
AMT	VB_APT	**0.0007**	41.37–49.12	43.25	**0.03**	1
AMT	C_APT	0.47	29–42.62	34		
HRS-D	VB_APT	**0.004**	2.5–12	10	**0.04**	0.41
HRS-D	C_APT	**0.009**	9–12.5	12		
TMT-A	VB_APT	**0.0007**	30.5–64.5	55	**0.01**	0.77
TMT-A	C_APT	**0.01**	56.5–139.5	76		
TMT-B	VB_APT	**0.0007**	82–215	152	0.12	0.71
TMT-B	C_APT	0.40	155–257.5	189		
TMT-BA	VB_APT	**0.007**	42–159	72	0.25	1.36
TMT-BA	C_APT	**0.002**	155–257.5	189		

Legend: MoCA (Montreal Cognitive Assessment); AMT (Attention Matrices Test); Trail Making Test (TMT-A; TMT-B; TMT A-B); HRS-D (Hamilton Rating Scale for Depression); Conventional Attention Processes Training (C_APT); Virtual Based-Attention Processes Training (VB_APT). ES (Effect Size) corresponds to between-groups analysis. ES: 0.2 = small effect; 0.5 = medium effect; 0.8 = large effect [41].

## Data Availability

Data will be available on-demand to the corresponding author.
